# ZNF283, a Krüppel-associated box zinc finger protein, inhibits RNA synthesis of porcine reproductive and respiratory syndrome virus by interacting with Nsp9 and Nsp10

**DOI:** 10.1186/s13567-023-01263-w

**Published:** 2024-01-15

**Authors:** Heyou Yi, Ruirui Ye, Ermin Xie, Lechen Lu, Qiumei Wang, Shaojun Wang, Yankuo Sun, Tao Tian, Yingwu Qiu, Qianwen Wu, Guihong Zhang, Heng Wang

**Affiliations:** 1 Guangdong Provincial Key Laboratory of Zoonosis Prevention and Control, College of Veterinary Medicine, South China Agricultural University, Guangzhou, 510462 China; 2 Key Laboratory of Animal Pathogen Infection and Immunology of Fujian Province, College of Animal Sciences, Fujian Agriculture and Forestry University, Fuzhou, 350002 China; 3 Maoming Branch, Guangdong Laboratory for Lingnan Modern Agriculture, Maoming, 525000 China; 4 National Engineering Research Center for Breeding Swine Industry, South China Agricultural University, Guangzhou, 510642 China

**Keywords:** Porcine reproductive and respiratory syndrome virus, Krüppel-associated box zinc finger protein, ZNF283, antiviral

## Abstract

Porcine reproductive and respiratory syndrome virus (PRRSV) is a viral pathogen with substantial economic implications for the global swine industry. The existing vaccination strategies and antiviral drugs offer limited protection. Replication of the viral RNA genome encompasses a complex series of steps, wherein a replication complex is assembled from various components derived from both viral and cellular sources, as well as from the viral genomic RNA template. In this study, we found that ZNF283, a Krüppel-associated box (KRAB) containing zinc finger protein, was upregulated in PRRSV-infected Marc-145 cells and porcine alveolar macrophages and that ZNF283 inhibited PRRSV replication and RNA synthesis. We also found that ZNF283 interacts with the viral proteins Nsp9, an RNA-dependent RNA polymerase, and Nsp10, a helicase. The main regions involved in the interaction between ZNF283 and Nsp9 were determined to be the KRAB domain of ZNF283 and amino acids 178–449 of Nsp9. The KRAB domain of ZNF283 plays a role in facilitating Nsp10 binding. In addition, ZNF283 may have an affinity for the 3' untranslated region of PRRSV. These findings suggest that ZNF283 is an antiviral factor that inhibits PRRSV infection and extend our understanding of the interactions between KRAB-containing zinc finger proteins and viruses.

**Supplementary Information:**

The online version contains supplementary material available at 10.1186/s13567-023-01263-w.

## Introduction

Porcine reproductive and respiratory syndrome virus (PRRSV) is responsible for sow abortion, boar sperm abnormalities, and respiratory disease in piglets [1]. Despite the abundant literature on the antigenic and genomic diversity, persistent infection, and immunosuppression of PRRSV, further investigations are warranted regarding virus‒host interactions.

PRRSV is classified within the family Arteriviridae and is characterized as a single-stranded positive-sense RNA virus with a genome length of approximately 15 kb and a minimum of 11 identified open reading frames (ORFs) [2]. The viral replicase polyproteins pp1a and pp1ab are crucial components of viral replication and are encoded by ORF1a and ORF1b, respectively. These polyproteins undergo proteolytic cleavage, resulting in the formation of 16 nonstructural proteins (Nsps) [3]. ORF2a and ORF3–ORF5 encode four envelope proteins, namely, GP2a, GP3, GP4, and GP5, which undergo N-glycosylation. ORF2b and ORF6 encode two glycosylated proteins (E and M) associated with the membrane. ORF7 encodes a nucleocapsid protein [4].

Efficient and accurate formation of replication and transcription complexes (RTCs) is essential for the synthesis of PRRSV RNA within the cellular environment. This process occurs on intracellular membranes that undergo remodelling to facilitate protein‒protein interactions and establish a conducive milieu for replication [5]. As in other positive-sense RNA viruses, virus-encoded hydrophobic membrane-spanning Nsps are frequently used to execute this mechanism [6]. The core components of PRRSV RTCs include Nsp9, Nsp10, Nsp11, and Nsp12 [7–9]. Nsp9, an RNA-dependent RNA polymerase, interacts with host proteins, including DEAD-box RNA helicase 5 (DDX5), retinoblastoma protein (Rb), annexin A2 (ANXA2), interleukin-2 enhancer-binding factor 2 (ILF2), nucleotide-binding oligomerization domain-like receptor X1 (NLRX1) and zinc finger antiviral protein (ZAP), to regulate viral replication [10–15]. PRRSV Nsp10 encodes an RNA helicase responsible for synthesizing subgenomic mRNA (sgmRNA) and potentially facilitating the unwinding of RNA secondary structures during replication [16]. The translocation of DDX18 from the nucleus to the cytoplasm is induced by Nsp10, ultimately facilitating viral proliferation [17]. Nsp9 and Nsp10 have been demonstrated to serve as the principal virulence factors of highly pathogenic Chinese PRRSV (HP-PRRSV) [18]. The primary function of Nsp11 is to suppress the production of host type I interferons [19–22]. The Nsp12 protein is specific to arteriviruses [23]. The presence of Cys35 and Cys79 in Nsp12 is essential for the synthesis of sgmRNAs [8] and facilitates viral mRNA synthesis and replication through the recruitment of Hsp70 [24].

Krüppel-associated box containing zinc-finger proteins (KRAB-ZFPs) represent the most extensive group of transcriptional repressors encoded by the genomes of higher organisms [25]. KRAB-ZFP proteins are composed of a KRAB domain and multiple C2H2 zinc fingers. Specifically, the C2H2 zinc-finger domains of KRAB-ZFP are responsible for DNA binding, whereas the KRAB domain functions as a transcriptional repressor [26, 27]. The proposed functions attributed to members of the KRAB-ZFP family include transcriptional repression of the RNA polymerase I, II, and III promoters, as well as RNA binding and splicing. In addition, these family members play crucial roles in metabolism, proliferation, differentiation, apoptosis, and cancer [28, 29]. Increasing evidence suggests that KRAB-ZFPs are involved in host antiviral defence mechanisms. For instance, the transcriptional activity of murine leukaemia viruses is suppressed by ZFP809 and ZFP998 [30, 31]. Moreover, SZF1 and ZNF557 can induce gene silencing of multiple genes associated with Epstein–Barr virus and Kaposi’s sarcoma-associated herpes virus [32, 33]. These studies demonstrated that KARB-ZFPs regulate viral replication through their interaction with viral nucleic acids. However, whether KRAB-ZFP controls viral replication by targeting viral proteins is unclear.

ZNF283 consists of a KRAB domain and 16 C2H2 zinc finger domains. Given the involvement of ZNF283 in the interplay between foot-and-mouth disease virus and its host [34], we investigated the potential involvement of ZNF283 in PRRSV replication. In the present study, PRRSV infection upregulated ZNF283 mRNA and protein expression, and ZNF283 impeded virus production and viral RNA synthesis. Furthermore, ZNF283 engages in interactions with Nsp9 and Nsp10 and specifically targets the PRRSV 3' untranslated region (UTR). Our findings indicate that the interplay between these proteins may be important for the regulation of PRRSV RNA synthesis.

## Materials and methods

### Cells and virus

The Marc-145 cell line derived from African green monkey kidneys and the HEK-293 T cell line derived from human embryonic kidneys were cultured in Dulbecco’s modified Eagle’s medium (Gibco, C11995500) supplemented with 10% foetal bovine serum (VivaCell Biosciences, 2238253). The cells were cultured under controlled conditions in an incubator at 37 °C with 5% CO_2_. The PRRSV strains XH-GD (GenBank accession number: EU624117.1), GM2-like (lineage 3) and NADC30-like (lineage 1) were preserved in our laboratory. The PRRSV JXA1 strain (GenBank accession no. EF112445.1) was obtained from Prof. Kegong Tian.

### Antibodies and reagents

The following primary antibodies were used: PRRSV N protein antibody (JNT, China; no. JN0401), mouse anti-glyceraldehyde-3-phosphate dehydrogenase (anti-GAPDH) antibody (TransGen Biotech, China; no. HC301), rabbit anti-HA (Cell Signaling Technology, USA; no. 3724 T), rabbit anti-Flag (Abmart, China; no. T20008M), mouse anti-HA (Abmart; no. M20003M), mouse anti-Flag (Beyotime, China; no. AF519), rabbit anti-GFP (Proteintech; no. 50430-2-AP), mouse anti-GFP (Proteintech; no. 66002-Ig), rabbit anti-β-actin (Proteintech; no. 81115-1-RR) and mouse anti-dsRNA (Scicons, Hungary; no. 10010500). The anti-ZNF283 antibody was generated by immunizing rabbits with bacterially expressed full-length porcine ZNF283.

### Plasmid construction and transfection

cDNA encoding ZNF283 was amplified from PK-15 cells and cloned and inserted into the pCAGGS vector with a C-terminal HA tag to construct a recombinant plasmid (pHA-ZNF283) using seamless cloning technology. The EGFP-tagged ZNF283 KRAB domain (amino acids 56–97) was generated by PCR amplification of pHA-ZNF283, which was cloned and inserted into the expression vector pEGFP-C1. PRRSV Nsp9, Nsp10, Nsp11, and Nsp12 were cloned and inserted into a pCAGGS vector with a C-terminal Flag tag to generate recombinant plasmids (pFlag-Nsp9, pFlag-Nsp10, pFlag-Nsp11, and pFlag-Nsp12). The truncations of pHA-ZNF283 and pFlag-Nsp9 were subcloned from the pHA-ZNF283 and pFlag-Nsp9 plasmids, respectively. The primers used for amplification are listed in Table 1. The expression plasmids were introduced into Marc-145 cells using Lipofectamine 3000 (Thermo Fisher Scientific, USA) and into HEK-293 T cells using polyethylenimine linear (PEI) (FuShen, China; no. FSF0002) according to the manufacturer’s instructions.

**Table 1:** **The primers used in the present study**

Primer	Sequence (5′-3′^a^)	Usage
HA-ZNF283-F	atcattttggcaaagATGAGCCTCTGGGCCCA	Amplification of ZNF283 and 1–191/296/408/527
HA-ZNF283-R	tgaaccgcctccaccTAAAGTTTCAGCAGCATCGACTGTCTCA	Amplification of ZNF283 and 192–663
HA-ZNF283 1–191-R	tgaaccgcctccaccAATGTAGGGTTTCTCTTGCTTATGAATAC	Amplification of ZNF283 1–191
HA-ZNF283 1–296-R	tgaaccgcctccaccTTCATAAGACTTTATGCCTATATGAATTTTCTGATG	Amplification of ZNF283 1–296
HA-ZNF283 1–408-R	tgaaccgcctccaccATGGATTCTCTCATGCTGAACCA	Amplification of ZNF283 1–408
HA-ZNF283 1–527-R	tgaaccgcctccaccTCCGTAGGGCCTCTCACC	Amplification of ZNF283 1–527
HA-ZNF283 192–663-F	atcattttggcaaagATGTGTCAGGAATGTGAGAAGGCTGG	Amplification of ZNF283 192–663
EGFP-ZNF283 56–97-F	gtaccgcgggcccgggatccTCGGTGACCTTCAAGGATGTG	Amplification of ZNF283 56–97
EGFP-ZNF283 56–97-R	tcagttatctagatccggtgctaATCCAGCGAGACCAAGTTGC	
Flag-Nsp9-F	atcattttggcaaagATGTTTAAACTGCTAGCCGCCAGC	Amplification of Nsp9 and 1–177/449
Flag-Nsp9-R	tgaaccgcctccaccCTCATGATTGGACCTGAGTTTTTCCC	Amplification of Nsp9 and 178/450–643
Flag-Nsp9 1–177-R	tgaaccgcctccaccGCTTCCAGTGTCACTGGGG	Amplification of Nsp9 1–177
Flag-Nsp9 178–449-F	atcattttggcaaagATGCCGGTGCACGCGG	Amplification of Nsp9 178–449/643
Flag-Nsp9 178–449-R	tgaaccgcctccaccGCCACCTCTCTTAGTCACCGC	Amplification of Nsp9 1/178–449
Flag-Nsp9 450–643-F	atcattttggcaaagATGCTGTCGTCTGGCGACCC	Amplification of Nsp9 450–643
Flag-Nsp10-F	atcattttggcaaagATGGGGAAGAAGTCCAGAATGTGCG	Amplification of Nsp10
Flag-Nsp10-R	tgaaccgcctccaccTTCCAAGTCTGCGCAAATAGCG	
Flag-Nsp11-F	atcattttggcaaagATGGGGTCGAGCTCCCCG	Amplification of Nsp11
Flag-Nsp11-R	tgaaccgcctccaccTTCAAGTTGAAAATAGGCCGTCTTGTCT	
Flag-Nsp12-F	atcattttggcaaagATGGGCCGCCATTTTACCTGG	Amplification of Nsp12
Flag-Nsp12-R	tgaaccgcctccaccATTCAGGCCTAAAGTTGGTTCAATGAC	

^a^The DNA sequences homologous to the vector region are depicted with lowercase letters, and the gene-specific regions are depicted with uppercase letters.

### shRNA-mediated knockdown

For short hairpin RNA (shRNA)-mediated knockdown of monkey ZNF283, three sequences targeting ZNF283 and a scrambled sequence were synthesized (Genewiz) (Table 2). The control scrambled shRNA sequence was used as the experimental control and theoretically had no effect on any gene. A pair of annealed oligonucleotides was phosphorylated and annealed using T4 polynucleotide kinase (New England Biolabs, USA; no. M0201S) and ligated into the AgeI/EcoRI-digested pLKO.1-U6-EF1a-mCherry-T2A-puro vector using T4 DNA ligase (Thermo Scientific, USA; no. EL0011).

**Table 2:** **The shRNA sequences used in the present study**

shRNA	Sequence (5'-3'^a^)
shmZNF283-1-F	ccggGCATCCATCTTCAGAAATAATCTCGAGATTATTTCTGAAGATGGATGCTTTTTG
shmZNF283-1-R	aattcAAAAAGCATCCATCTTCAGAAATAATCTCGAGATTATTTCTGAAGATGGATGC
shmZNF283-2-F	ccggGCCTCGCTAAACATGAGATAACTCGAGTTATCTCATGTTTAGCGAGGCTTTTTG
shmZNF283-2-R	aattcAAAAAGCCTCGCTAAACATGAGATAACTCGAGTTATCTCATGTTTAGCGAGGC
shmZNF283-3-F	ccggGGCCTTTGGTAGTGGCTATCACTCGAGTGATAGCCACTACCAAAGGCCTTTTTG
shmZNF283-3-R	aattcAAAAAGGCCTTTGGTAGTGGCTATCACTCGAGTGATAGCCACTACCAAAGGCC
scrambled shRNA-F	ccggGAAGAGGACACGCCTTAGACTCTCGAGAGTCTAAGGCGTGTCCTCTTCTTTTTG
scrambled shRNA-R	aattcAAAAAGAAGAGGACACGCCTTAGACTCTCGAGAGTCTAAGGCGTGTCCTCTTC

^a^Flanking sequences (lowercase letters) represent the AgeI/EcoRI restriction sites. shRNA target sequences are shown in uppercase letters.

For determination of the effects of ZNF283 knockdown on PRRSV replication and transcription, Marc-145 cells in 6-well plates were transfected with shmZNF283 or scrambled shRNA. The cells were infected with PRRSV at 24 h after transfection and subsequently harvested at specific time intervals for subsequent analyses.

### Subcellular proteome extraction

Marc-145 cells were transfected with HA-ZNF283 for 24 h and then mock-infected or infected with PRRSV for 36 h. The isolation of nuclear components was conducted by employing a nuclear and cytoplasmic protein extraction kit (Beyotime, no. P0027). The cells were detached using a cell scraper and collected by centrifugation at 1000 rpm. Subsequently, the cells were treated with 200 μL of cytoplasmic protein extraction buffer A containing the protease inhibitor phenylmethylsulfonyl fluoride for 15 min on ice and then added to 10 μL of buffer B. After vigorous vortex mixing and centrifugation at 13 000 rpm for 10 min at 4 °C, the supernatants were collected for analysis of cytoplasmic proteins. The precipitates were resuspended in 50 μL of nuclear protein extraction buffer for 30 min on ice and centrifuged at 13 000 rpm for 15 min at 4 ℃. The supernatants were collected for nuclear protein analysis. SDS‒PAGE and Western blotting analysis were performed on the supernatant.

### Western blotting

The cells were collected using NP-40 lysis buffer (Beyotime, no. P0013F) with the protease inhibitor phenylmethylsulfonyl fluoride (Beyotime, no. ST506). Subsequently, equivalent quantities of proteins were separated using sodium dodecyl sulphate–polyacrylamide gel electrophoresis and subsequently transferred onto a nitrocellulose membrane (Millipore). The membranes were blocked using a 5% skim milk solution and incubated overnight at 4 °C with the designated primary antibodies. Following three washes with Tris-buffered saline containing Tween-20, the membranes were incubated with IRDye 800CW goat anti-mouse IgG (H + L) or anti-rabbit IgG (H + L) for 1 h at room temperature. Subsequently, the membranes were subjected to further washing and visualized utilizing an azure laser scanner (USA).

### Viral titres

Marc-145 cells were cultured in 96-well plates, and the virus supernatants were subjected to tenfold serial dilution. A total of 100 μL of the diluted supernatant was added to each well, with eight replicates per well. After a 2 h incubation period at 37 °C, the medium was replaced with a novel medium comprising 2% foetal bovine serum. The plates were incubated for 3–5 days prior to the determination of virus titres. The cells were observed daily for cytopathic effects, and the determination of 50% tissue culture infective dose (TCID_50_) was conducted using the Reed–Muench method.

### RNA extraction and reverse transcription quantitative real-time PCR (RT‒qPCR)

Total cellular RNA was extracted using an RNA Rapid Extraction Kit (Fastagen, China; no. 220011), and 1 μg of RNA obtained from each sample was reverse transcribed using a reverse transcription kit (Accurate Biology, China; no. A0212) to generate cDNA. Subsequently, complementary DNA (cDNA) was utilized as a template in the ChamQ Universal SYBR qPCR master mix (Vazyme, China; no. Q711-02). The mRNA expression levels were normalized to the expression of GAPDH. All RT‒qPCR experiments were performed using a CFX96 real-time system (Bio-Rad, USA). The primers used for qPCR are listed in Table 3. The RT‒qPCR primer sequences used for PRRSV gRNA and sgmRNAs were previously published [35].

**Table 3:** **The primers employed in the present study**

Primer	Sequence (5′-3′^a^)	Usage
pGL3-5'UTR-F	cgggctcgagatctATGACGTATAGGTGTTGGCTCTATGC	Amplification of PRRSV 5'UTR
pGL3-5'UTR-R	cggaatgccaagcttGGTTAAAGGGGTGGAGAGACC	
pGL3-5'-3'UTR-F	aagatcgccgtgtaaTGGGCTGGCATTCTTTGG	Amplification of PRRSV 3'UTR
pGL3-5'-3'UTR-R	tcggtcgacggatccTTTTTTAATTGCGGCCGCATGGT	
pGL3-3'UTR-R	cgccccgactctagaTTTTTTAATTGCGGCCGCATGG	
q-mZNF283-F	TCCATGGCATAACTTGGGGAA	qRT-PCR for detection of Marc-145 ZNF283
q-mZNF283-R	TGGCCATGCATTACACAAACTG	
q-sZNF283-F	CGAGTTTGCCTGAGAAGAGCG	qRT-PCR for detection of PAM ZNF283
q-sZNF283-R	GAAGACACGGGAAAGGGACG	
q-mGAPDH-F	TGATGACATCAAGAAGGTGGTGAAG	qRT-PCR for detection of Marc-145 GAPDH
q-mGAPDH-R	TCCTTGGAGGCCATGTGGGCCAT	
q-sGAPDH-F	CCTTCCGTGTCCCTACTGCCAAC	qRT-PCR for detection of PAM GAPDH
q-sGAPDH-R	GACGCCTGCTTCACCACCTTCT	

^a^The DNA sequences homologous to vector regions are depicted in lowercase letters, and the gene-specific regions are shown in uppercase letters.

### Confocal microscopy

Marc-145 cells were cultured on glass coverslips in 12-well plates and subsequently transfected with the designated plasmids, as indicated in the figure legends. For visualization of endogenous immunofluorescence, Marc-145 cells were infected with PRRSV at a multiplicity of infection (MOI) of 0.2 and incubated at 37 °C. After 24 h of transfection or infection, the cells were washed with cold phosphate-buffered saline (PBS) and fixed with 4% paraformaldehyde for 15 min. Subsequently, the cells were permeabilized with 0.1% Triton X-100 in PBS for 30 min at room temperature and blocked with 5% bovine serum albumin (BSA) in PBS for 30 min. The cells were incubated with primary antibodies in 5% BSA in PBS at 4 °C overnight. The cells were then rinsed with PBS and incubated with Alexa Fluor 488 goat anti-mouse IgG (H + L) secondary antibodies (Invitrogen, USA; no. A11011) or Alexa Fluor 594 goat anti-rabbit IgG (H + L) (Invitrogen; no. A11012) for 1 h. Finally, the cells were washed and stained with 4′,6-diamidino-2-phenylindole (DAPI) (Beyotime; no. C1006) for 5 min. The cells were imaged using an inverted fluorescence microscope (Nikon Eclipse Ti2-U) or a confocal laser scanning microscope (Fluoviewver.10, Olympus, Japan). The analysis of signal overlap between various channels was conducted using FluoView FV10i software (Olympus). The scatterplot points aligned along the diagonal axis suggest colocalization, while the scatterplot points aligned along the x- and y-axes suggest the absence of colocalization.

### Coimmunoprecipitation (co-IP) assay

HEK-293T cells were cultured in 100 mm dishes and subsequently transfected or cotransfected with suitable eukaryotic expression plasmids. At 36 h post-transfection, the cells were collected and lysed on ice for 15 min in 1 mL of NP-40 lysis buffer containing the protease inhibitor phenylmethylsulfonyl fluoride. The resulting samples were then subjected to centrifugation at 12 000 rpm for 10 min at 4 °C. A fraction of the supernatant obtained from the lysed cells was used as the whole lysate sample, while the remaining fractions were immunoprecipitated with the specified antibodies or isotype control or with normal IgG overnight at 4 °C, followed by treatment with a protein A/G agarose gel (Santa Cruz Biotechnology, USA; no. SC-2003) for 6 h at 4 °C. The beads were washed thrice with 1 mL of NP-40 lysis buffer. Finally, the whole lysate samples and immunoprecipitation samples were resuspended in sodium dodecyl sulphate–polyacrylamide gel electrophoresis loading buffer (EpiZyme, LT101S), heated to 95 °C for 8 min, and analysed via Western blotting.

### Dual-luciferase reporter assay

Linear fragments of the PRRSV 5'UTR or 3'UTR were amplified via PCR from the PRRSV genome. Seamless cloning was used to construct the plasmid. The resulting 5'UTR fragments were cloned and inserted into the BglII/HindIII-digested pGL3-control vector (Promega) to generate the pGL3-5'UTR construct. Subsequently, the 3'UTR fragments were inserted into the pGL3-5'UTR plasmids at the XbaI and BamHI restriction sites, resulting in the pGL3-5'-3'UTR construct. Additionally, the 3'UTR fragments were cloned and inserted into the XbaI-digested pGL3-Luc vector, generating the pGL3-3'UTR construct. The primers used for amplification are listed in Table 3. Marc-145 cells were cultured in 12-well plates and transfected with 0.8 μg of HA-tagged ZNF283 plasmid or HA-tagged empty vector, 0.4 μg of reporter plasmid (pGL3-5'-3'UTR, pGL3-5'UTR, or pGL3-3'UTR), or 0.02 μg of pRL-TK reference plasmid using Lipofectamine 3000 according to the manufacturer’s instructions. The Dual-Luciferase Reporter Assay System (TransGen Biotech) was used to evaluate firefly and Renilla luciferase activities in lysed cells according to the manufacturer’s guidelines.

### Statistical analysis

Statistical analyses were conducted using GraphPad Prism 8 software with two-tailed unpaired t tests. Significant differences are denoted in the figures through asterisks as follows: * *P* < 0.05, ** *P* < 0.01, *** *P* < 0.001, and **** *P* < 0.0001.

## Results

### PRRSV infection activates ZNF283 expression and induces ZNF283 translocation from the nucleus to the cytoplasm

**Figure 1:**
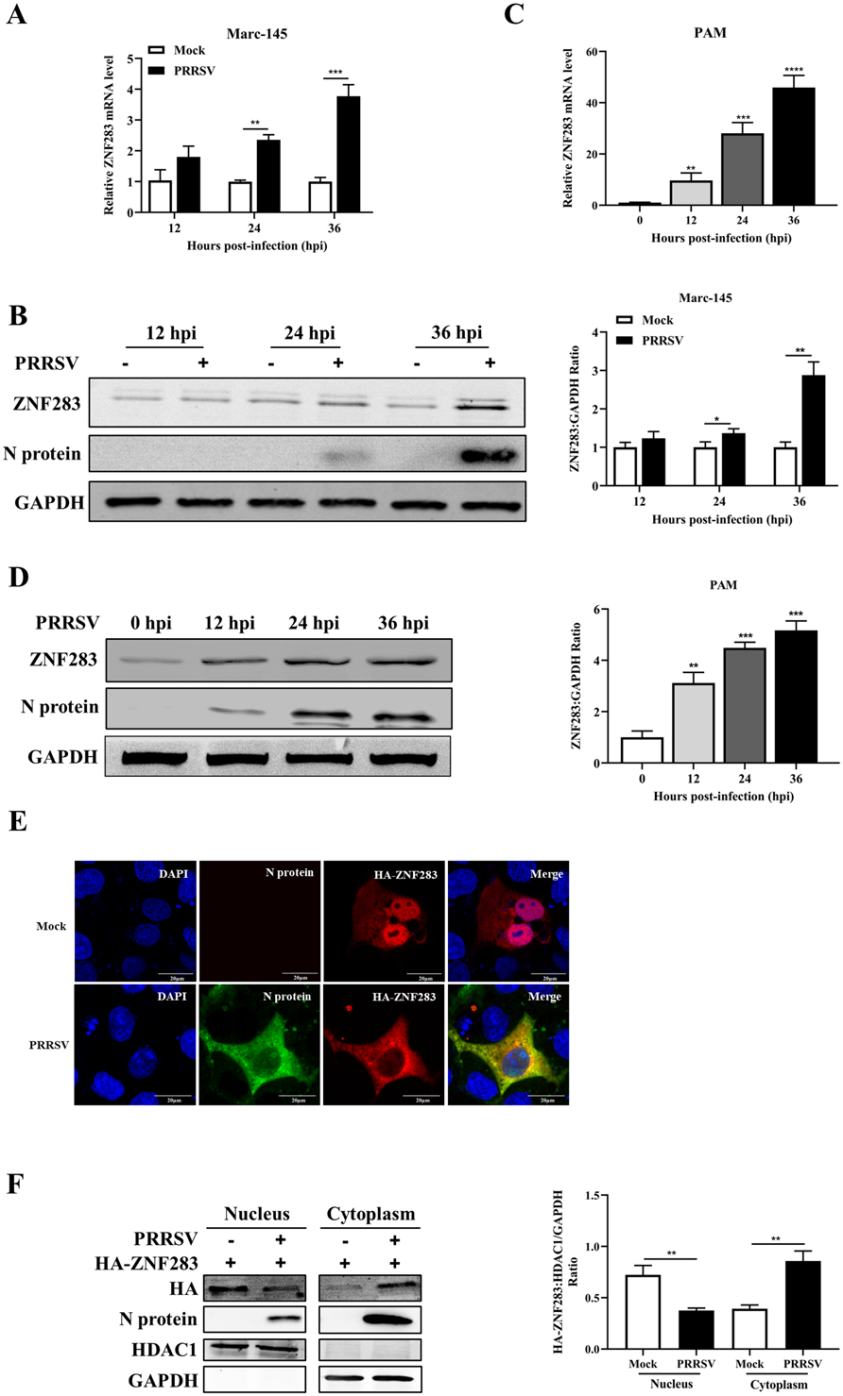
**The expression of ZNF283 is upregulated by PRRSV infection.** Marc-145 cells and PAMs were mock-infected or infected with PRRSV at an MOI of 0.2 for various durations. The cells were then collected and analysed for ZNF283 mRNA and protein expression levels using RT‒qPCR (**A** and** C**) and Western blotting (**B** and** D**), respectively. **E** Marc-145 cells were transfected with HA-tagged ZNF283 for 24 h and then infected with PRRSV at an MOI of 0.2. Mock-infected cells served as controls. At 24 h post-infection, the cells were fixed and immunostained with mouse anti-N protein and rabbit anti-HA antibodies. Nuclei were counterstained with DAPI. **F** Marc-145 cells were transfected with HA-ZNF283 for 24 h and then infected with PRRSV at an MOI of 0.2. At 36 h post-infection, cellular fractionation followed by Western blotting was performed. HDAC1 and GAPDH were used as control proteins for the nuclear and cytosolic fractions, respectively.

For analysis of the potential effects of PRRSV infection on ZNF283 expression, Marc-145 cells and porcine alveolar macrophages (PAMs) were infected with a low dose of PRRSV for various durations. The mRNA and protein levels of ZNF283 were measured by qPCR and Western blotting, respectively. The findings indicated that ZNF283 mRNA expression in Marc-145 cells and PAMs was elevated following PRRSV infection (Figures 1A and C), whereas ZNF283 protein levels were significantly increased compared to those in the uninfected mock-treated cells (Figures 1B and D). Both the mRNA and protein expression of ZNF283 significantly increased after infection with high-dose PRRSV (see Additional file 1). These results suggested that ZNF283 expression can be activated by viral infection.

To explore whether PRRSV infection affects the localization pattern of ZNF283, we conducted confocal microscopy assays in Marc-145 cells. In the absence of PRRSV, HA-ZNF283 exhibited primary nuclear localization, with a minor fraction of the protein being observed within the cytoplasm (Figure 1E). In addition, subcellular localization analysis of HA-ZNF283 was performed by nucleocytoplasmic separation. Consistent with the observations made through confocal microscopy, PRRSV-infected cells presented an evident reduction in HA-ZNF283 in the nuclear fraction, while HA-ZNF283 had a nuclear localization in noninfected cells (Figure 1F). These results indicated that PRRSV infection promoted the translocation of ZNF283 from the nucleus to the cytoplasm.

### ZNF283 overexpression inhibits PRRSV replication

**Figure 2:**
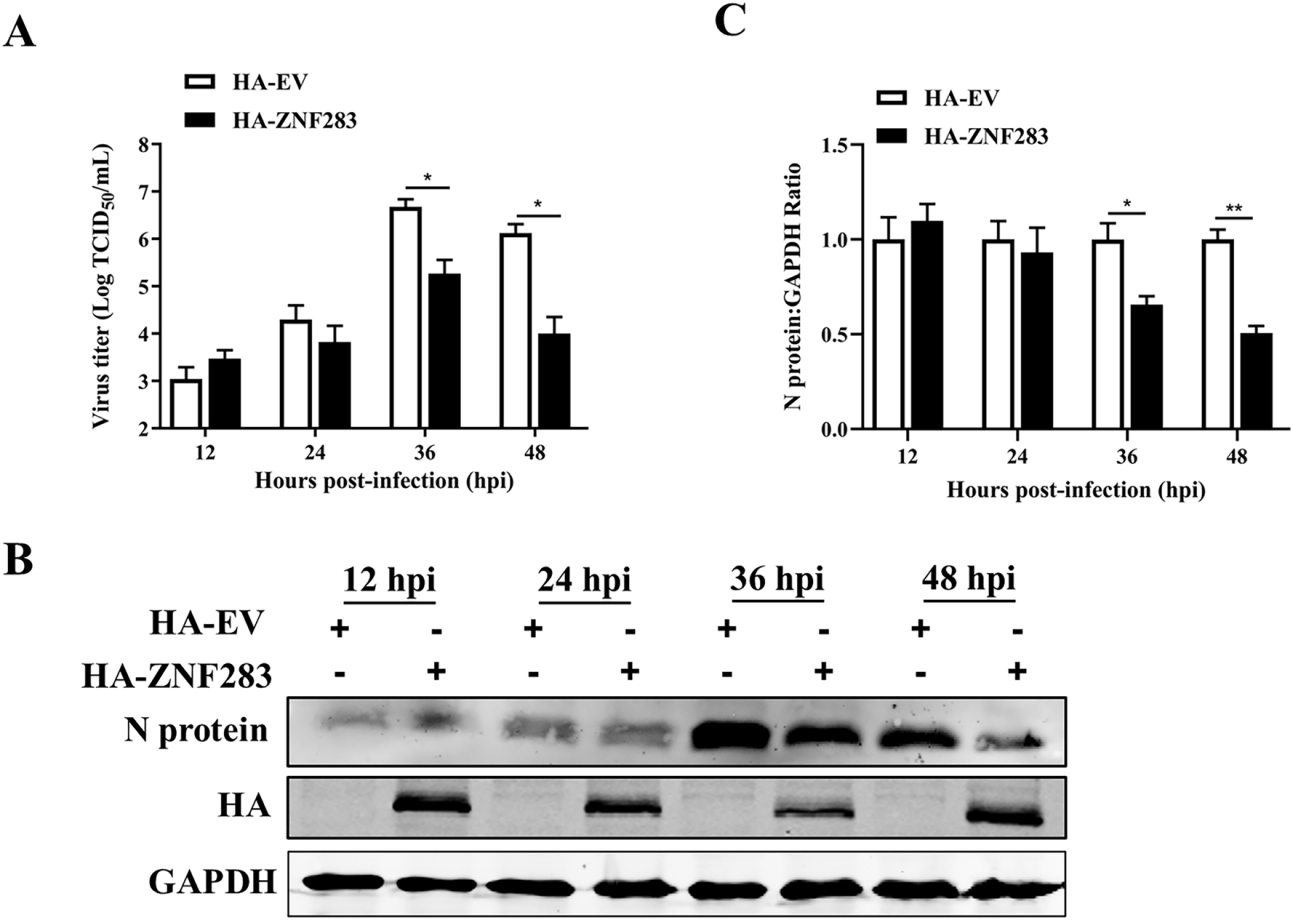
**Overexpression of ZNF283 inhibits PRRSV replication.** Marc-145 cells were transfected with HA-tagged ZNF283 or HA-tagged empty vectors for 24 h and then infected with PRRSV at an MOI of 0.2. At 12, 24, 36, and 48 h post-infection, viral titres in the cell supernatant (**A**) and the expression levels of the N protein (**B** and** C**) were assessed using TCID_50_ and Western blotting, respectively.

For analysis of the impact of ZNF283 on PRRSV replication, Marc-145 cells were transfected with either HA-tagged ZNF283 plasmids or HA-tagged empty vectors for 24 h before infection with PRRSV for various time periods. We found that upregulation of ZNF283 expression notably decreased the viral titre (Figure 2A) and the expression of the nucleocapsid (N) protein (Figures 2B, C) at 36 h and 48 h post-infection in Marc-145 cells. Moreover, ZNF283 inhibited the replication of various strains of PRRSV, such as GM2-like, JXA1 and NADC30-like strains (Additional file 2). These results suggested that ZNF283 suppressed the replication of PRRSV.

### ZNF283 knockdown promotes PRRSV replication

**Figure 3:**
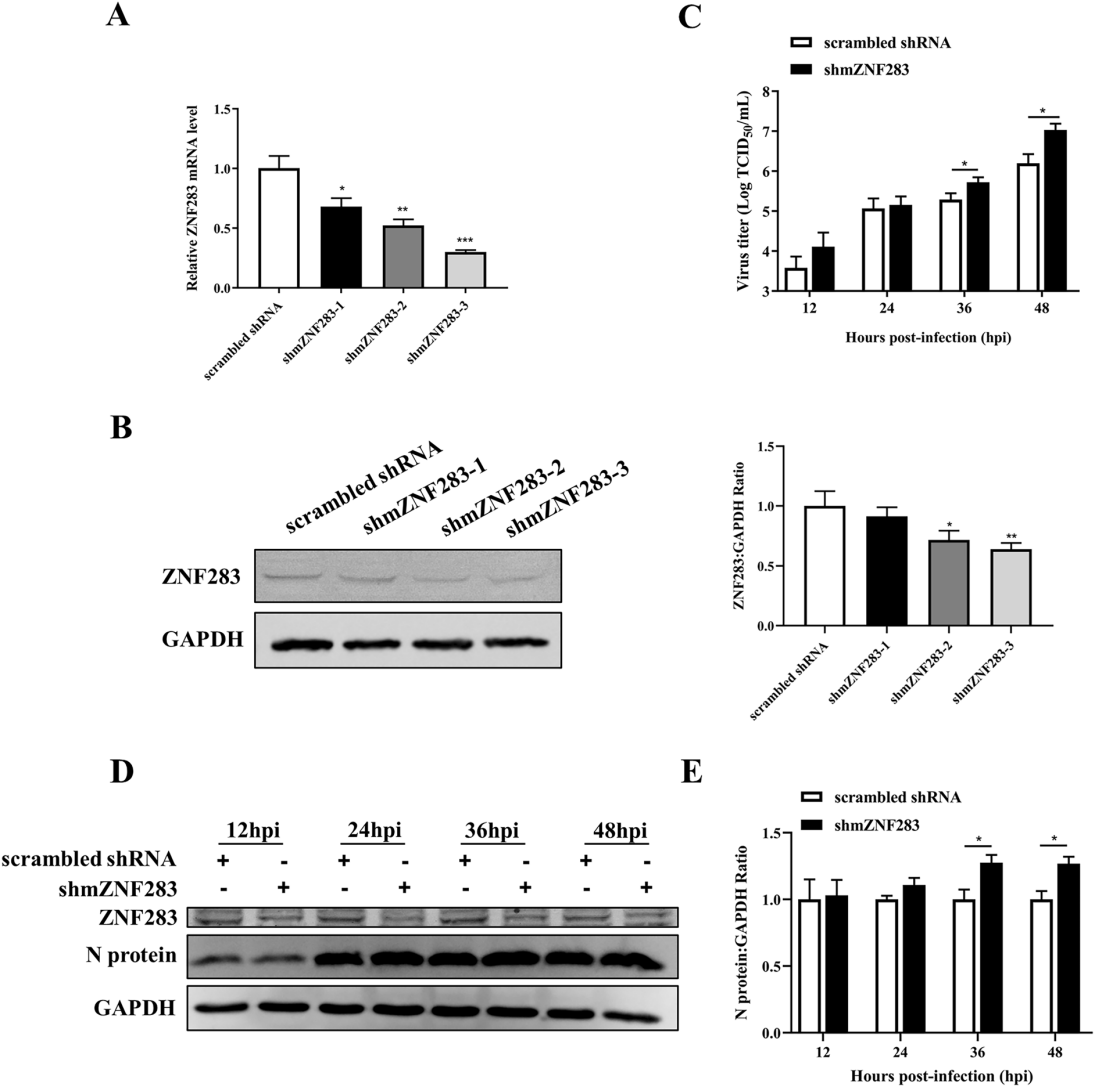
**ZNF283 knockdown facilitates the proliferation of PRRSV.** Marc-145 cells were transfected with three independent shRNAs targeting ZNF283 for 24 h, and RT‒qPCR (**A**) and Western blotting (**B**) were used to evaluate the knockdown efficiency of ZNF283. Marc-145 cells were transfected with shmZNF283 or scrambled shRNA for 24 h and then infected with PRRSV at an MOI of 0.5 for various time periods. The culture supernatant was used for the TCID_50_ assay (**C**), and N protein expression levels were analysed using Western blotting (**D** and ** E**).

To further confirm the role of ZNF283 in PRRSV infection, we designed an shRNA targeting the ZNF283 gene in Marc-145 cells, and its knockdown efficiency was determined using RT‒qPCR and Western blotting. At 24 h post-transfection, the transcript levels of ZNF283 were significantly impaired by all three independent shRNAs (Figure 3A). shmZNF283-3, which had the highest knockdown efficiency at protein levels, was selected for further experiments (Figure 3B). Marc-145 cells were transfected with shmZNF283-3 or scrambled shRNA and then infected with PRRSV for various durations. ZNF283 depletion significantly increased viral titres and N protein expression in Marc-145 cells throughout the infection process (Figures 3C, D and E). The results showed that the downregulation of ZNF283 facilitated PRRSV replication.

### ZNF283 suppresses the synthesis of PRRSV subgenomic and genomic RNA

**Figure 4:**
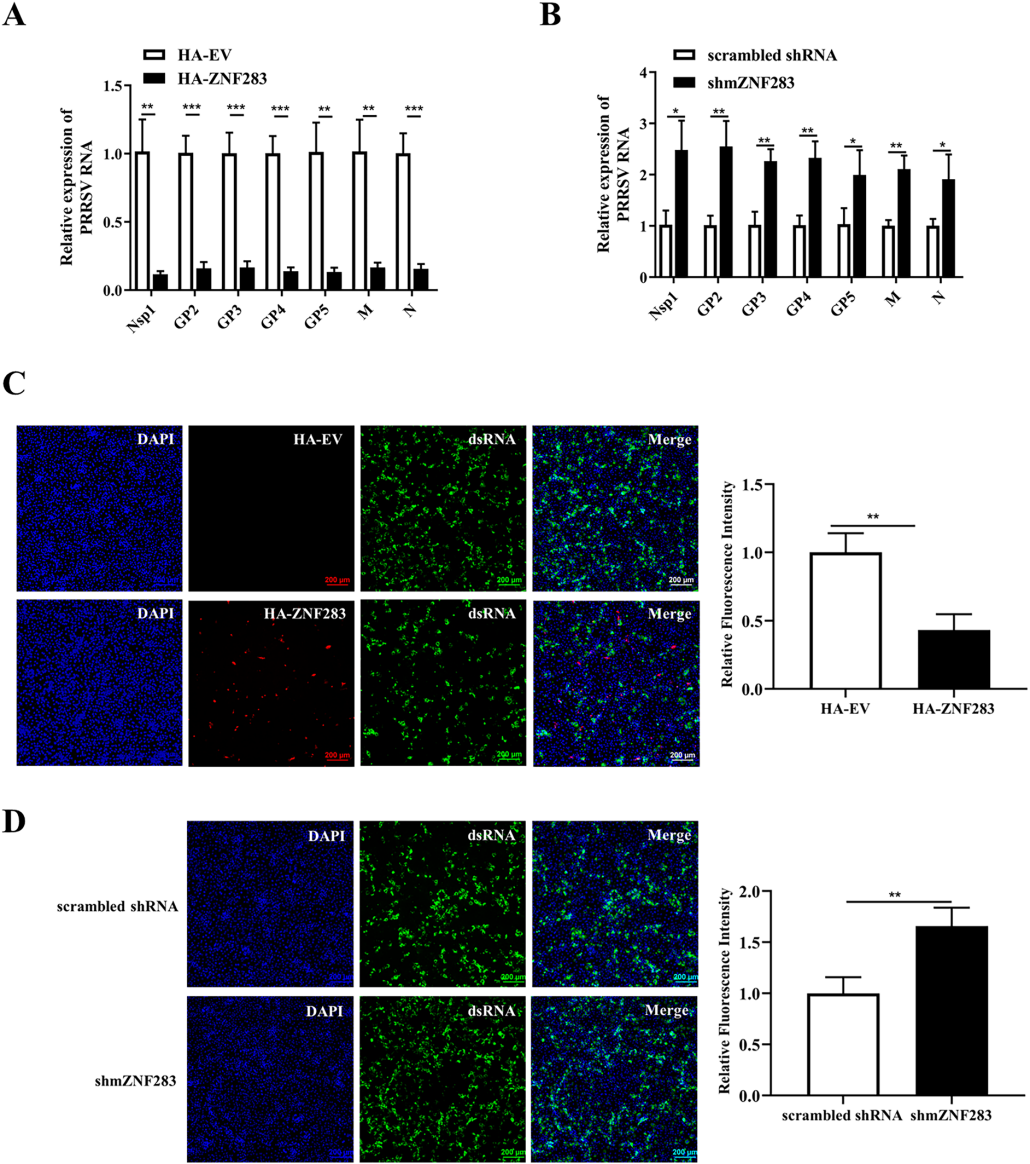
**ZNF283 suppresses PRRSV RNA synthesis.** Marc-145 cells were transfected with HA-tagged ZNF283 or shmZNF283 and then infected with PRRSV at an MOI of 0.2. An empty vector or scrambled shRNA was used as a control. **A** and **B** Cells were harvested at 36 h post-infection for RT‒qPCR analysis of viral RNA expression. **C** and **D** At 36 h post-infection, the cells were fixed for immunofluorescence staining of dsRNA (green). The nuclei were counterstained with DAPI (blue).

For analysis of the potential involvement of ZNF283 in PRRSV RNA synthesis, Marc-145 cells were transfected with either HA-tagged ZNF283 or an HA-tagged vector for 24 h before infection with PRRSV for 36 h. The Nsp1, GP2, GP3, GP4, GP5, M and N genes represent the expression levels of the viral genome, sgmRNA2, sgmRNA3, sgmRNA4, sgmRNA5, sgmRNA6 and sgmRNA7, respectively. Compared to those in the control group, the expression of ZNF283 effectively suppressed the production of the Nsp1, GP2, GP3, GP4, GP5, M and N genes (Figure 4A). ZNF283 overexpression significantly decreased the level of dsRNA, an intermediate in viral genome replication (Figure 4C). Furthermore, transfection with the shmZNF283-3 plasmid resulted in the downregulation of ZNF283 in Marc-145 cells, leading to significant upregulation of the expression of the Nsp1, GP2, GP3, GP4, GP5, M and N genes (Figure 4B). ZNF283 knockdown significantly elevated the level of dsRNA (Figure 4D). These results suggested that ZNF283 exerts a detrimental effect on PRRSV RNA synthesis.

### ZNF283 interacts with PRRSV Nsp9 and Nsp10

**Figure 5:**
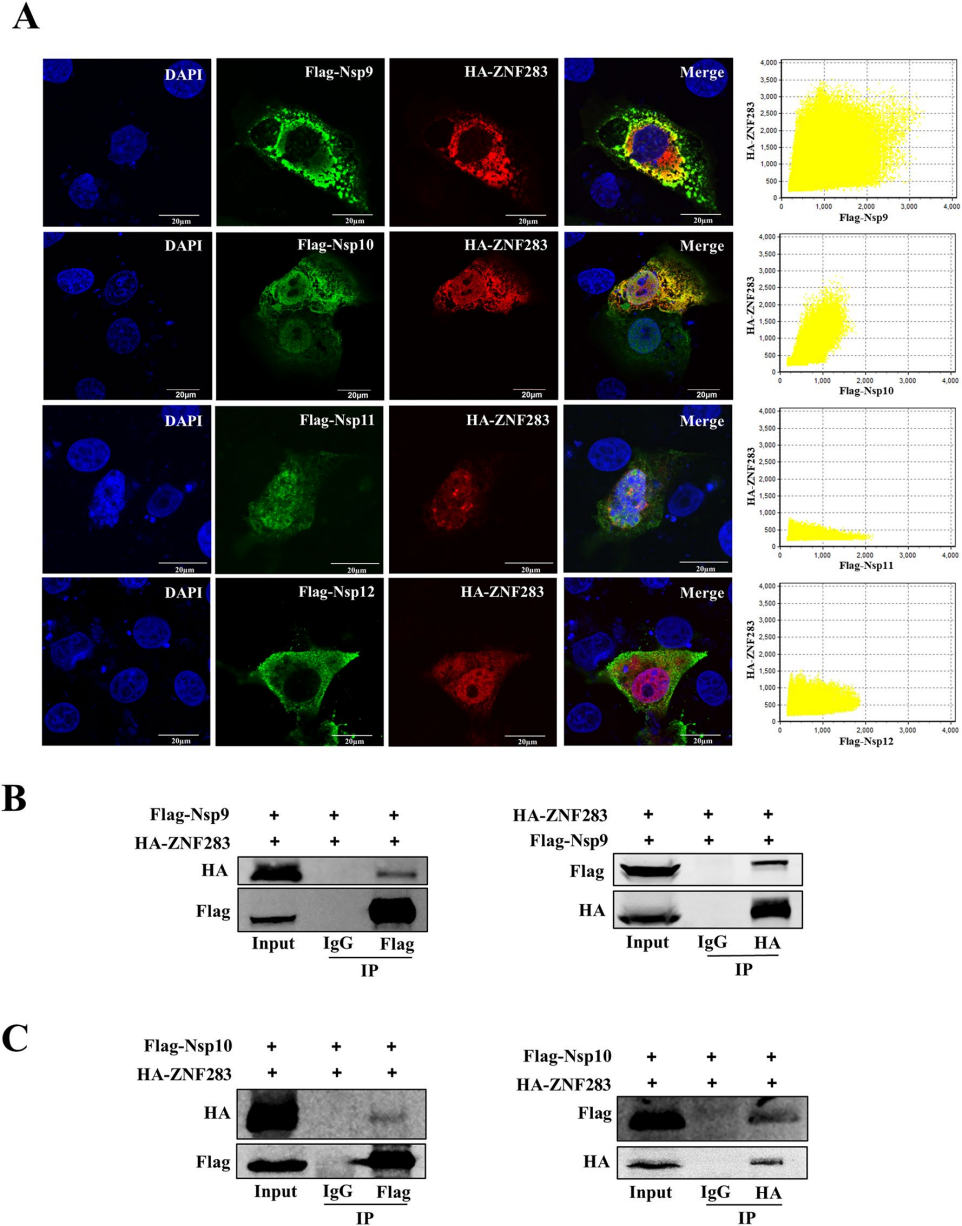
**ZNF283 interacts with PRRSV Nsp9 and Nsp10. A** Marc-145 cells were cotransfected with HA-tagged ZNF283 and plasmid-encoded Flag-tagged PRRSV viral proteins (Nsp9, Nsp10, Nsp11, and Nsp12) for 24 h. The cells were then fixed, and immunofluorescence analysis was performed using rabbit anti-HA and mouse anti-Flag antibodies. Nuclei were stained with DAPI. **B** and** C** HEK-293T cells were cotransfected with HA-tagged ZNF283 and Flag-tagged Nsp9 or Nsp10 for 36 h, followed by immunoprecipitation with mouse anti-Flag or mouse anti-HA antibodies. Cell lysates were analysed by Western blotting using rabbit anti-HA or rabbit anti-Flag antibodies.

KRAB-ZFPs constitute the most extensive group of transcriptional regulators in mammalian cells [36]. The core components of the PRRSV RTC are primarily Nsp9, Nsp10, Nsp11, and Nsp12. For determination of which transcription complex protein interacted with ZNF283, the HA-tagged ZNF283 plasmid was transfected into Marc-145 cells along with the Flag-tagged Nsp9, Nsp10, Nsp11, and Nsp12 plasmids. Colocalization of the ZNF283 and PRRSV proteins was observed by confocal microscopy. ZNF283 was intensely colocalized with Nsp9 and Nsp10 in the cytoplasm but was not colocalized with Nsp11 or Nsp12 (Figure 5A). Immunoprecipitation assays revealed that ZNF283 interacted with Nsp9 and Nsp10 (Figures 5B, C).

### The KRAB domain of ZNF283 interacts with the Nsp9 amino acid (aa) 178–449 region

**Figure 6:**
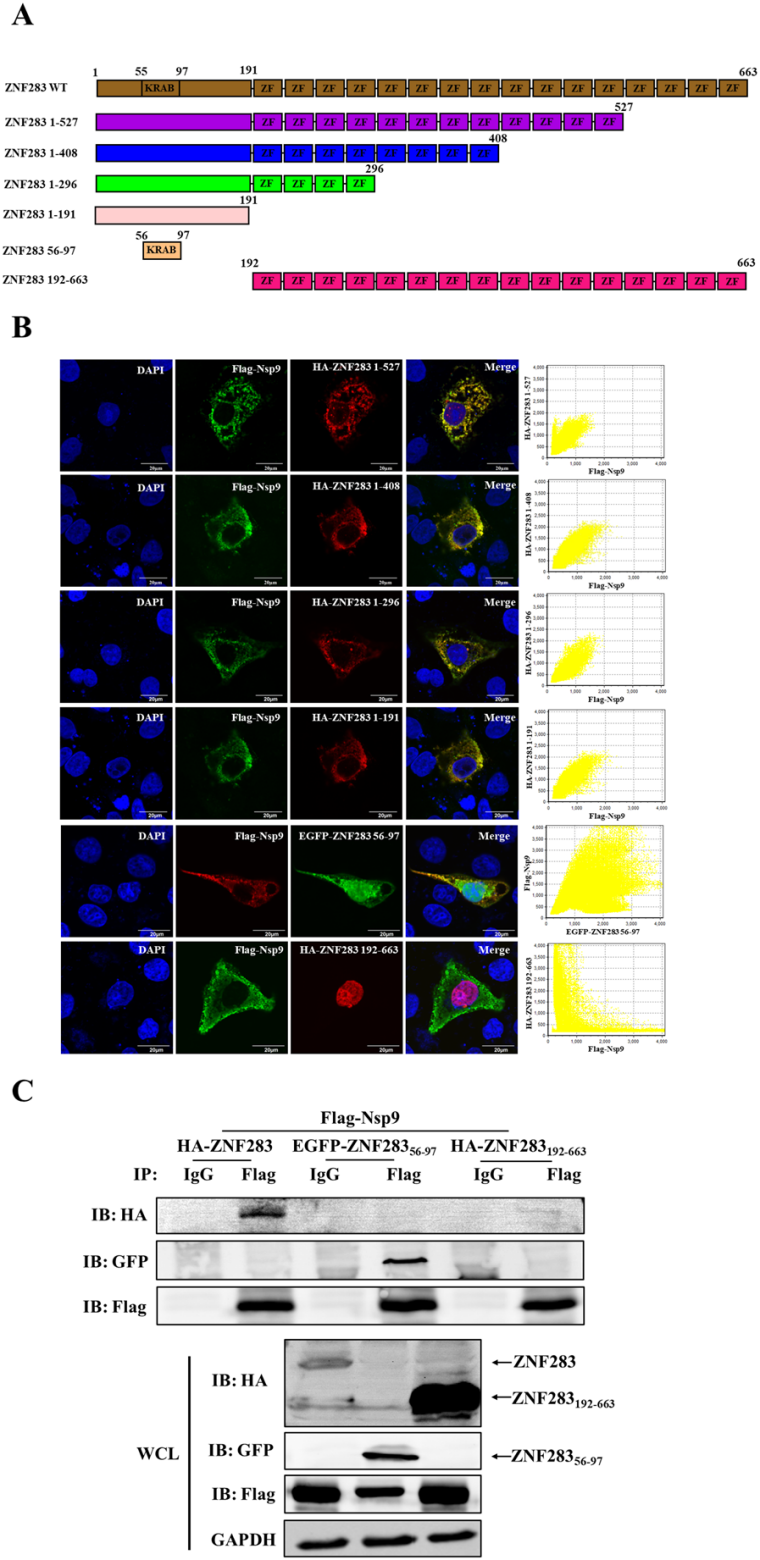
**PRRSV Nsp9 interacts with the ZNF283 KRAB domain from aa 56–97. A** Diagrammatic illustration of diverse fragments of ZNF283. **B** Marc-145 cells were cotransfected with Flag-tagged Nsp9 and plasmid-encoded HA-tagged ZNF283 truncations (aa 1–191, 1–296, 1–408, 1–520, and 192–663) or EGFP-tagged ZNF283 aa 56–97 for 24 h. The cells were fixed and immunostained using either a mouse anti-Flag or rabbit anti-HA antibody. Nuclei were counterstained with DAPI. **C** HEK-293T cells were cotransfected with the plasmids Flag-tagged Nsp9 and full-length HA-tagged ZNF283, EGFP-tagged ZNF283 truncations (aa 56–97), or HA-tagged ZNF283 truncations (aa 192–663) for 36 h. Following cell lysis, immunoprecipitation analysis was conducted using a mouse anti-Flag antibody.

ZNF283 consists of a KRAB domain (aa 56–97) and 16 C2H2 zinc finger motifs (aa 192–663). Based on the ZNF283 structure, a series of plasmids expressing truncated mutants of HA-tagged ZNF283 were generated, encompassing aa 1–191, 1–296, 1–408, 1–527, and 192–663 (Figure 6A). Western blotting also revealed that the ZNF283 truncation mutants were stably expressed (Additional file 3). Marc-145 cells were cotransfected with Flag-tagged Nsp9 and truncated ZNF283 plasmids and incubated for 24 h. Confocal microscopy revealed that Nsp9 was located within the cytoplasm and that the ZNF283 mutants, which encompassed aa 1–191, exhibited colocalization with Nsp9 (Figure 6B). Further investigation confirmed that aa 56–97 of ZNF283 colocalized with Nsp9 within the cytoplasm, whereas aa 192–663 was confined to the nucleus and did not colocalize with Nsp9 (Figure 6B). Co-IP analysis revealed that Nsp9 interacted with aa 56–97 of ZNF283, whereas no interaction was observed with aa 192–663 (Figure 6C).

**Figure 7:**
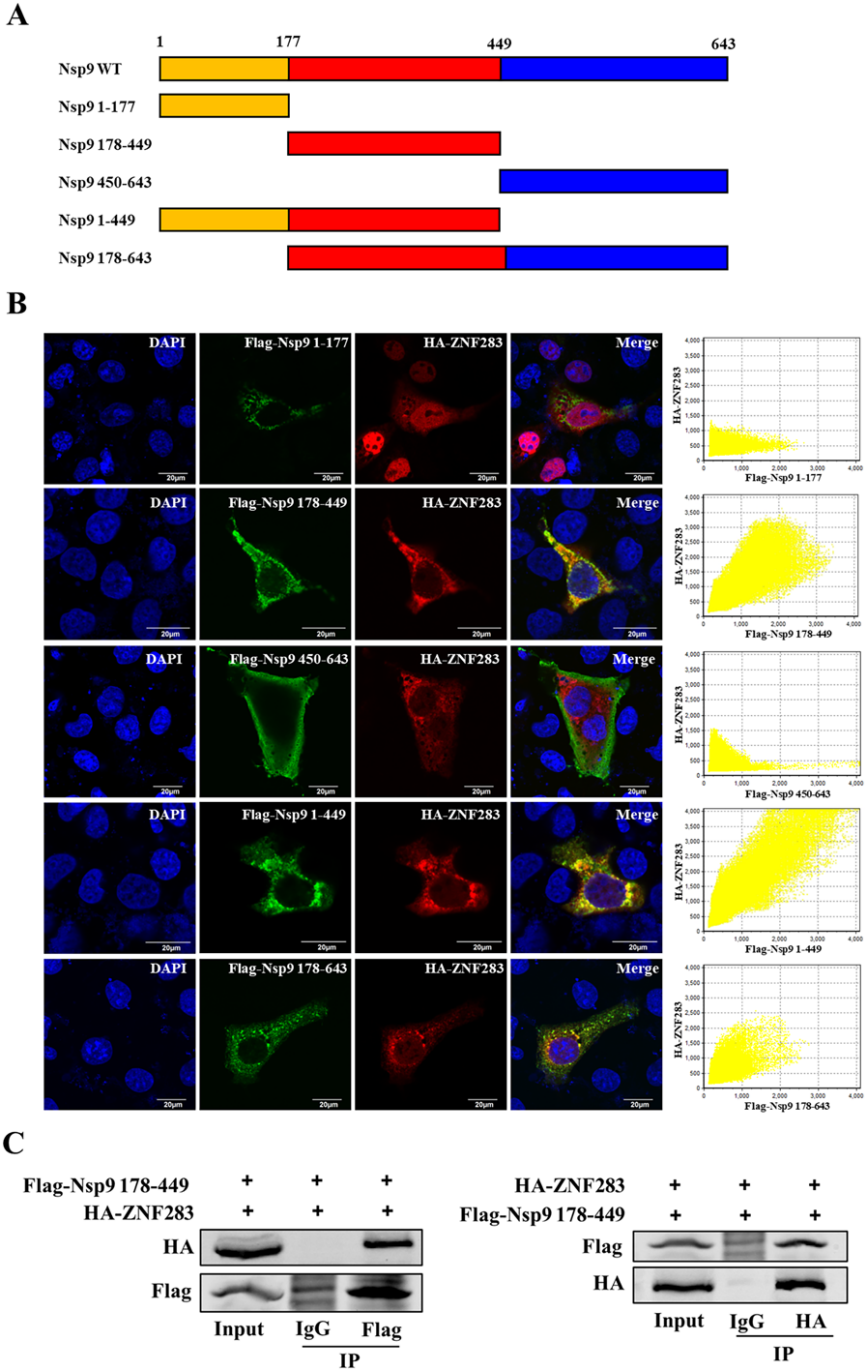
**ZNF283 interacts with the aa 178–449 region of Nsp9. A** Schematic representation of Nsp9 truncation mutants. **B** Marc-145 cells were cotransfected with HA-tagged ZNF283 and plasmid-encoded Flag-tagged Nsp9 truncations (aa 1–177, 178–449, 450–643, 1–499, and 178–643) for 24 h. The cells were fixed and immunostained with mouse anti-Flag and rabbit anti-HA antibodies. Nuclei were detected by counterstaining with DAPI. **C** HEK-293T cells were cotransfected with the HA-tagged ZNF283 plasmid and Flag-tagged truncated Nsp9 (aa 178–449) for 36 h. Immunoprecipitation of cell lysates was performed using either mouse anti-Flag or mouse anti-HA antibodies, followed by Western blotting analysis using either rabbit anti-HA or rabbit anti-Flag antibodies.

Based on the predicted domain of Nsp9 [15], five Flag-tagged Nsp9 truncation mutant plasmids were constructed containing aa 1–449, 178–643, 1–177, 178–449, and 450–643 (Figure 7A). The results of the Western blotting analysis demonstrated that the Nsp9 truncation mutants exhibited stable expression (Additional file 4). HA-tagged ZNF283 and Flag-tagged Nsp9 truncation mutant plasmids were cotransfected into Marc-145 cells. Confocal microscopy revealed that the regions from Nsp9 aa 178–449, 1–449, and 178–643 colocalized with ZNF283, whereas the regions from aa 1–177 and 450–643 did not (Figure 7B). Hence, an interaction likely occurs between Nsp9 aa 178–449 and ZNF283. The results of the co-IP assay demonstrated a significant interaction between Nsp9 aa 178–449 and ZNF283 (Figure 7C).

**Figure 8:**
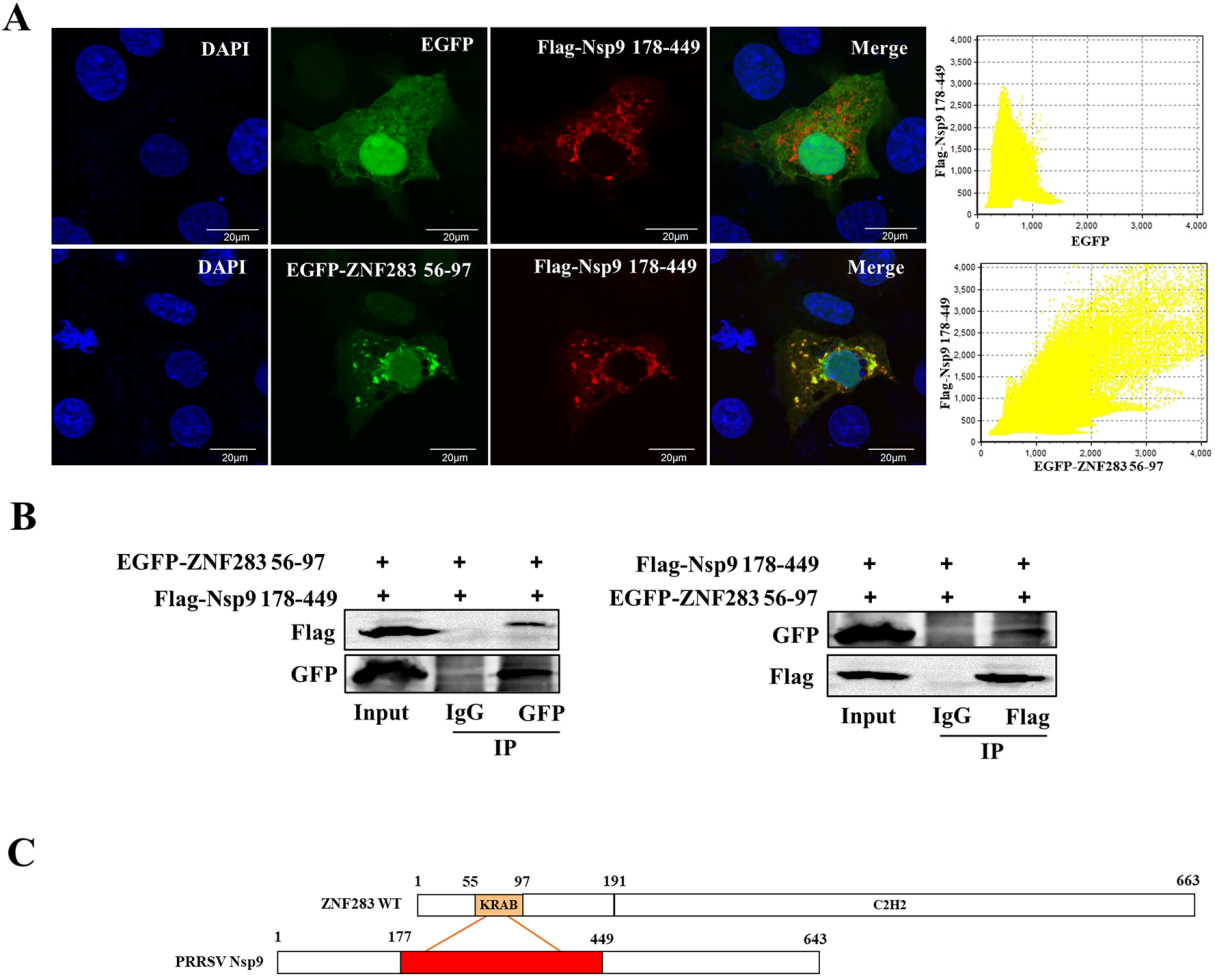
**The KRAB domain of ZNF283 interacts with the aa 178–449 region of Nsp9. A** Marc-145 cells were cotransfected with Flag-tagged truncated Nsp9 spanning aa 178–449 and EGFP-tagged ZNF283 KRAB domain spanning aa 56–97 or an EGFP-tagged vector. After 24 h, the cells were stained with DAPI and incubated with antibodies specific for the Flag-tagged protein. **B** HEK-293T cells were cotransfected with Flag-tagged truncated Nsp9 (aa 178–449) and EGFP-tagged ZNF283 aa 56–97. Thirty-six hours after transfection, the cell lysate was immunoprecipitated with mouse anti-Flag or mouse anti-GFP antibodies and then immunoblotted using the indicated antibodies from different species. **C** Schematic representation of the interaction between ZNF283 and PRRSV Nsp9.

To verify whether ZNF283 aa 56–97 interacted with Nsp9 aa 178–449, we cotransfected plasmids encoding EGFP-tagged ZNF283 aa 56–97 and Flag-tagged Nsp9 aa 178–449 into Marc-145 or HEK-293 T cells. The EGFP-tagged ZNF283 aa 56–97 truncation mutant was expressed normally (Additional file 3). In Marc-145 cells, the two proteins were strongly colocalized in the cytoplasm (Figure 8A). Furthermore, HEK-293 T cell lysates were incubated with GFP beads or Flag beads, and the proteins were examined by Western blotting using anti-GFP or anti-Flag antibodies. As shown in Figure 8B, ZNF283 (aa 56–97) coimmunoprecipitated with Nsp9 (aa 178–449). These findings collectively demonstrated that the interaction between ZNF283 and Nsp9 depends on aa 56–97 (KRAB domain) of ZNF283 and aa 178–449 of Nsp9 (Figure 8C).

### The KRAB domain of ZNF283 interacts with Nsp10

**Figure 9:**
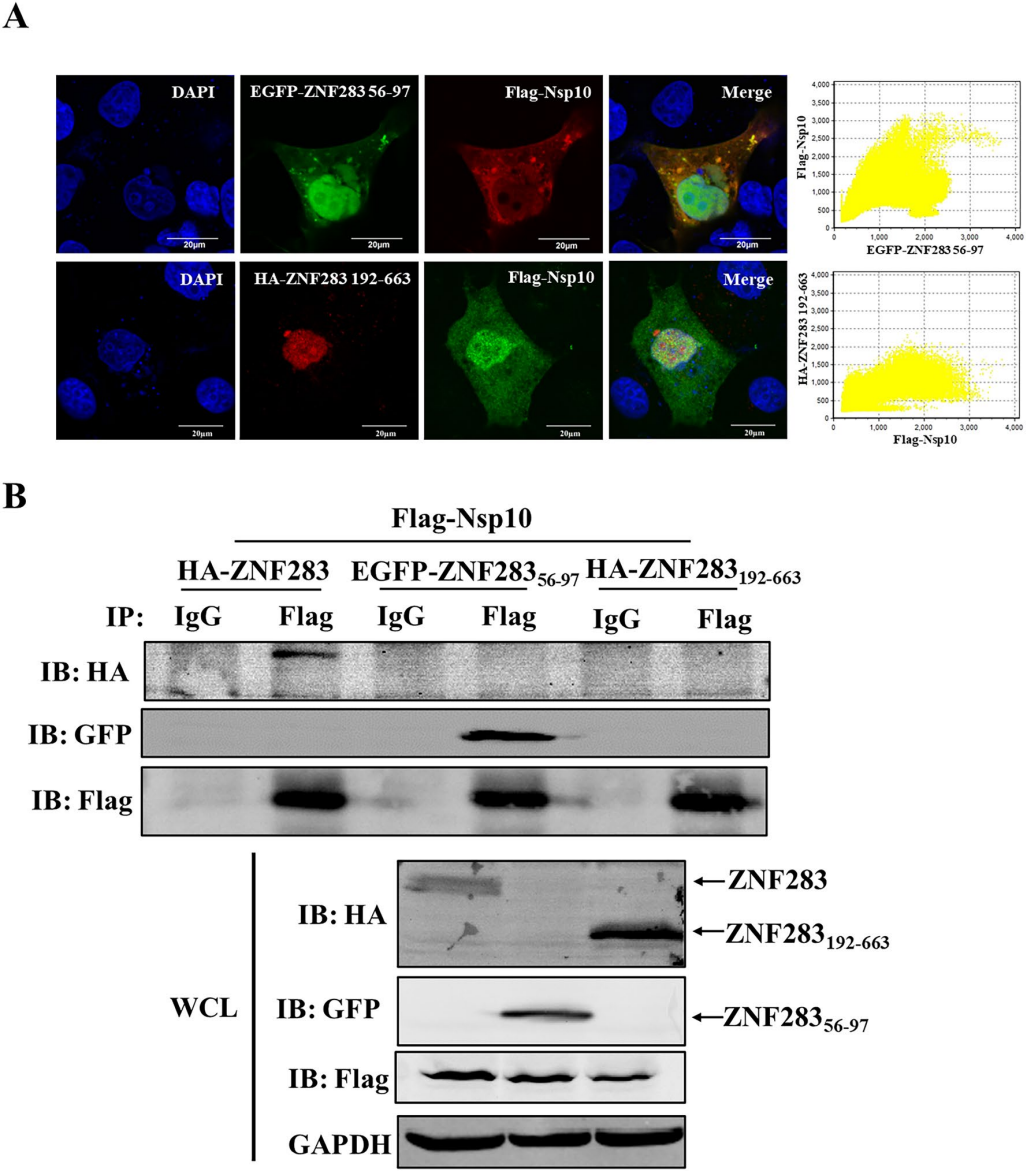
**PRRSV Nsp10 interacts with the ZNF283 KRAB domain from aa 56–97. A** Marc-145 cells were cotransfected with Flag-tagged Nsp10 and EGFP-tagged ZNF283 truncations (aa 56–97) or HA-tagged ZNF283 truncations (aa 192–663) for 24 h. The cells were fixed and immunostained using either mouse anti-Flag or rabbit anti-HA antibodies. Nuclei were counterstained with DAPI. **B** HEK-293T cells were cotransfected with Flag-tagged Nsp10 and HA-tagged ZNF283, EGFP-tagged ZNF283 truncations (aa 56–97), or HA-tagged ZNF283 truncations (aa 192–663) for 36 h. After cell lysis, immunoprecipitation analysis was performed using a mouse anti-Flag antibody.

For determination of the primary domain of ZNF283 that interacts with Nsp10, Marc-145 or HEK-293 T cells were cotransfected with the EGFP-ZNF283 aa 56–97 or HA-ZNF283 aa 192–663 plasmids along with Flag-Nsp10. Confocal microscopy revealed that ZNF283 aa 56–97 colocalized with Nsp10 within the cytoplasm, whereas ZNF283 aa 192–663 displayed nuclear localization and did not colocalize with Nsp10 (Figure 9A). Co-IP further demonstrated that Nsp10 interacted with the aa 56–97 region of ZNF283 but not with the aa 192–663 region (Figure 9B). These results suggested that the interaction between ZNF283 and Nsp10 occurred via the KRAB domain.

### ZNF283 specifically targets the PRRSV 3'UTR

**Figure 10:**
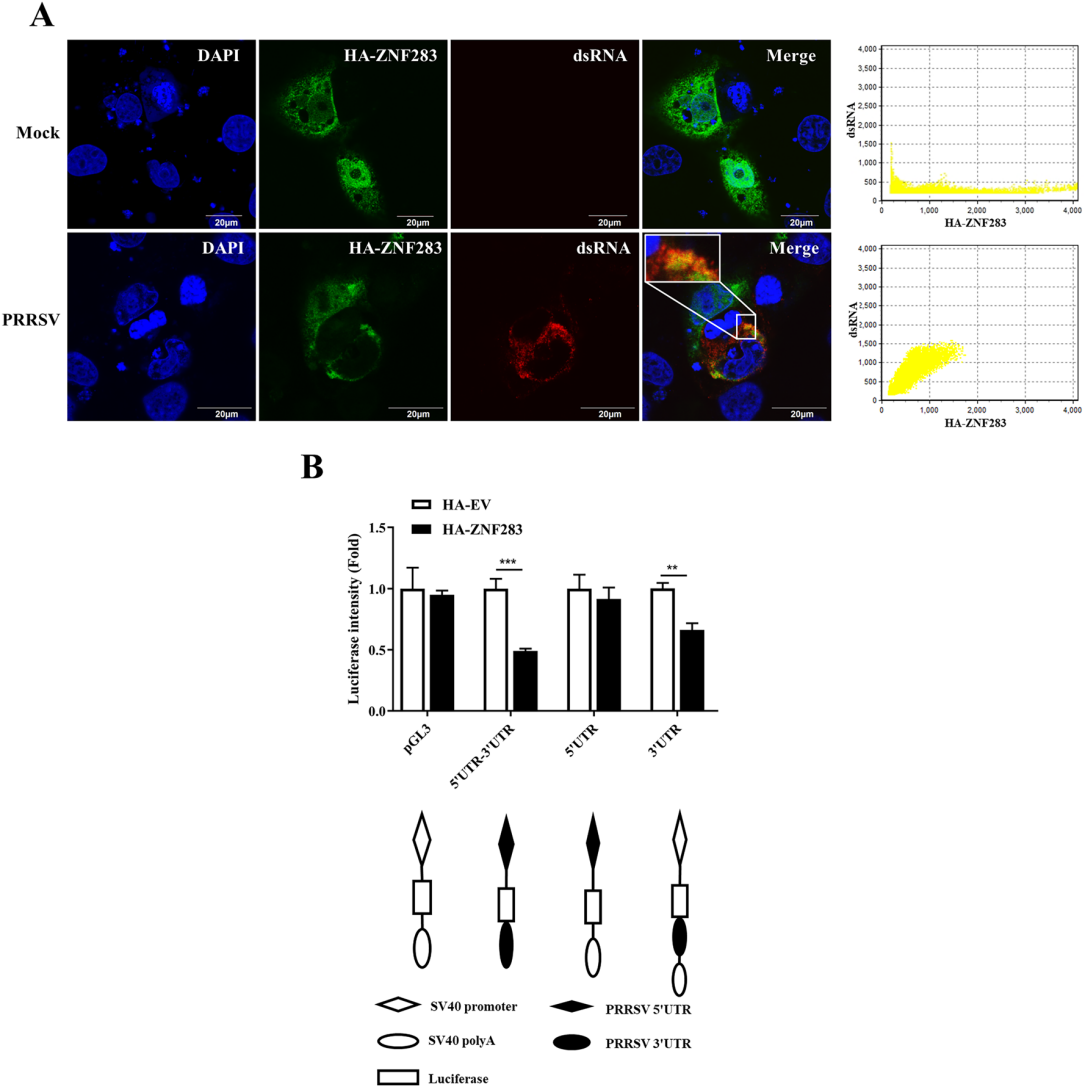
**ZNF283 has a specific affinity for the PRRSV 3'UTR. A** Marc-145 cells were transfected with HA-tagged ZNF283 for 24 h and then mock-infected or infected with PRRSV at an MOI of 0.2 for 36 h. The cells were fixed for immunofluorescence staining with HA-tagged (green) and dsRNA (red). The nuclei were labelled with DAPI. **B** The 5'UTR or 3'UTR of PRRSV was cloned and inserted into the control reporter pGL3-Luc, as indicated. The resulting constructs, HA-tagged ZNF283 and pRL-TK, which are plasmids expressing Renilla luciferase and were used to normalize transfection efficiency, were cotransfected into Marc-145 cells. After 24 h of transfection, the cells were lysed, and luciferase activities were quantified.

We previously demonstrated that ZNF283 specifically prevents the accumulation of PRRSV RNA, suggesting that ZNF283 targets specific viral RNA sequences. In light of the dsRNA replication intermediates generated during PRRSV replication, we investigated the potential colocalization of ZNF283 with PRRSV dsRNA. Confocal microscopy demonstrated colocalization between ZNF283 and the viral dsRNA within the infected cells (Figure 10A).

The 5'UTR and 3'UTR are important regulatory elements in the process of virus replication and transcription [37–39]. To identify the target sequence of ZNF283 in the PRRSV sequence, we cloned the PRRSV 5'UTR or 3'UTR into a pGL3-Luc reporter using a previously described strategy [40] and analysed the effect of ZNF283 on the PRRSV 5'UTR and 3'UTR-mediated luciferase activity. The constructs were evaluated using Marc-145 cells. Similarly, compared with the control, ZNF283 significantly reduced luciferase activity when both the 5'UTR and 3'UTR or the 3'UTR alone were fused; however, ZNF283 did not significantly suppress 5'UTR luciferase activity (Figure 10B). These findings suggested that the ZNF283 target sequence was located within the PRRSV 3'UTR. Furthermore, these findings suggested that the target sequence of ZNF283 was functional when it was inserted downstream of the luciferase coding sequence.

## Discussion

PRRSV is an important pathogen with a substantial impact on the global swine industry. However, the intricacies of the interactions between PRRSV and host proteins remain unclear. In the present study, ZNF283 overexpression effectively inhibited PRRSV replication. Additionally, ZNF283 disrupts viral RNA synthesis and establishes interactions with Nsp9 and Nsp10.

PRRSV possesses multiple Nsps within the replicases ORF1a and ORF1b, which actively engage in the synthesis of PRRSV RNA. Notably, viral Nsp9 and the RNA helicase Nsp10 play important roles in this process [41, 42]. ZAP, alternatively referred to as ZC3HAV1 or PARP13, possesses four consecutive CCCH-type zinc finger motifs at its N-terminus and has demonstrated efficacy as a broad-spectrum antiviral protein. ZAP has been found to inhibit various viruses from the Alphaviridae [43], Filoviridae [44], Retroviridae [45], and other related families. It has been reported that ZAP can also inhibit the replication of PRRSV and that the zinc finger domain aa 1–240 of ZAP interacts with PRRSV Nsp9 aa 150–160 [15]. Our research revealed that the KRAB-containing zinc finger protein ZNF283, which is known for its ability to inhibit PRRSV replication and RNA synthesis, interacts with Nsp9 and Nsp10 via its KRAB domain (aa 56–97) rather than its zinc finger domain. This interaction may play an important role in regulating PRRSV RNA synthesis.

KRAB-ZFPs bind to DNA or RNA through their zinc finger region, and their repressive function arises from the KRAB-mediated recruitment of KAP1 [KRAB-associated protein 1, also known as tripartite motif protein 28 (TRIM28)] [46–48]. The zinc finger protein ZNF809 inhibits the transcription of murine leukaemia virus by recruiting the KAP1 protein to the primer-binding site [30]. ZNF304 facilitates the recruitment of KAP1 to the 5'-long terminal repeats of human immunodeficiency virus-1 (HIV-1), resulting in the suppression of viral transcription [49]. We speculated that ZNF283 may augment the inhibitory effect on PRRSV RNA synthesis through the recruitment of KAP1. However, further investigation is needed to validate this hypothesis.

The 5'UTRs and 3'UTRs of numerous positive-sense RNA viruses contain specific signals for RNA synthesis that play important roles in viral replication. Examples of these viruses include mouse hepatitis virus [50, 51], bovine viral diarrhoea virus [52, 53], and PRRSV [37, 38]. The PRRSV 5’UTR has eukaryotic promoter activity, and the conserved stem‒loop 2 is critical for sgmRNA synthesis [38, 54]. The 3'UTR of PRRSV, which encompasses an RNA pseudoknot interaction between two terminal stem‒loop structures, has been identified as a molecular switch involved in viral RNA synthesis [55]. The zinc finger protein is capable of binding to the 5'UTR or 3'UTR of the virus through its zinc finger domain. ZAP interacts with RNA and recruits exosome complexes to selectively degrade viral RNAs [56, 57], including the 5'UTR of the Nef segment of HIV-1 [57], 3'-long terminal repeats of Moloney murine leukaemia virus [40], and 3'UTR of xenotropic murine leukaemia virus-related virus [58]. Our findings demonstrated that ZNF283 suppresses luciferase activity mediated by the PRRSV 3'UTR. Additionally, we observed the colocalization of ZNF283 with the intermediate dsRNA involved in PRRSV RNA synthesis. These observations suggest a potential interaction between ZNF283 and the PRRSV 3'UTR, although further investigation is needed to substantiate this speculation.

In conclusion, our study demonstrates that ZNF283 functions as a highly effective cell-intrinsic antiviral factor against PRRSV replication, potentially through its interaction with viral Nsp9 and Nsp10. Notably, we identified the KRAB domain of ZNF283 and the aa 178–449 region of Nsp9 as critical interaction regions. ZNF283 binds to Nsp10 via its KRAB domain. These findings strongly contribute to our understanding of the antiviral response of the host to PRRSV infections.

### Supplementary Information

**Additional file 1. ZNF283 expression is upregulated by PRRSV infection**. Marc-145 cells and PAMs were mock-infected or infected with PRRSV at an MOI of 1 for various durations. The cells were then collected and analysed for ZNF283 mRNA and protein expression levels using RT‒qPCR (**A **and **C**) and Western blotting (**B** and **D**), respectively.

**Additional file 2. ZNF283 inhibits the replication of different PRRSV strains. **Marc-145 cells were transfected with HA-tagged empty vectors or HA-tagged ZNF283 for 24 h and then infected with different PRRSV strains (GM2-like, JXA1, and NADC30-like) at an MOI of 0.2. At 36 h post-infection, viral titres in the cell supernatant (**A**) and the expression levels of the N protein (**B**) were assessed using TCID_50_ and Western blotting, respectively.

**Additional file 3. Verification of the expression of ZNF283 truncation mutants. **HEK-293T cells were transfected with plasmid-encoded HA-tagged full-length and truncated ZNF283 (aa 1–191, 1–296, 1–408, 1–520, and 192–663) (**A**) or EGFP-tagged ZNF283 aa 56–97 (**B**) for 36 h. Cell lysates were analysed via Western blotting using rabbit anti-HA or rabbit anti-EGFP.

**Additional file 4. Verification of the expression of Nsp9 truncation mutants. **HEK-293T cells were transfected with empty vector or plasmid-encoded Flag-tagged full-length or truncated Nsp9 (aa 1–177, 178–449, 450–643, 1–499, or 178–643) for 36 h. Cell lysates were analysed via Western blotting using mouse anti-Flag.

## Data Availability

All the data generated or analysed during this study are included in the article.
