# Supplementary figures and images for: ZNF283, a Krüppel-associated box zinc finger protein, inhibits RNA synthesis of porcine reproductive and respiratory syndrome virus by interacting with Nsp9 and Nsp10
Source: Vet Res. 2024 Jan 15;55:9. doi: 10.1186/s13567-023-01263-w (PMC10790482; doi:10.1186/s13567-023-01263-w)

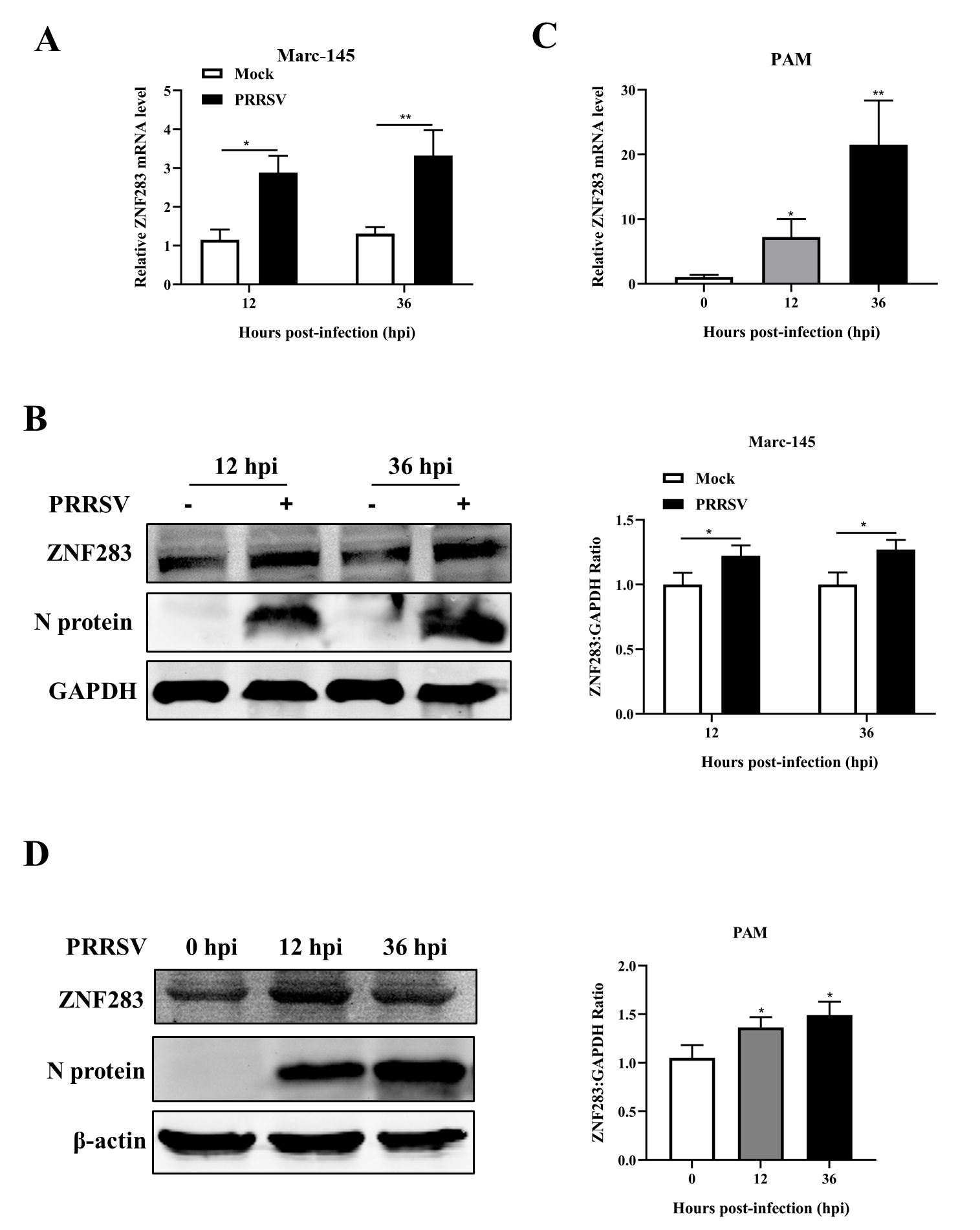

Supplement: Supplementary file 1 — Additional file 1. ZNF283 expression is upregulated by PRRSV infection. Marc-145 cells and PAMs were mock-infected or infected with PRRSV at an MOI of 1 for various durations. The cells were then collected and analysed for ZNF283 mRNA and protein expression levels using RT‒qPCR (A and C) and Western blotting (B and D), respectively. [file 13567_2023_1263_MOESM1_ESM.docx]

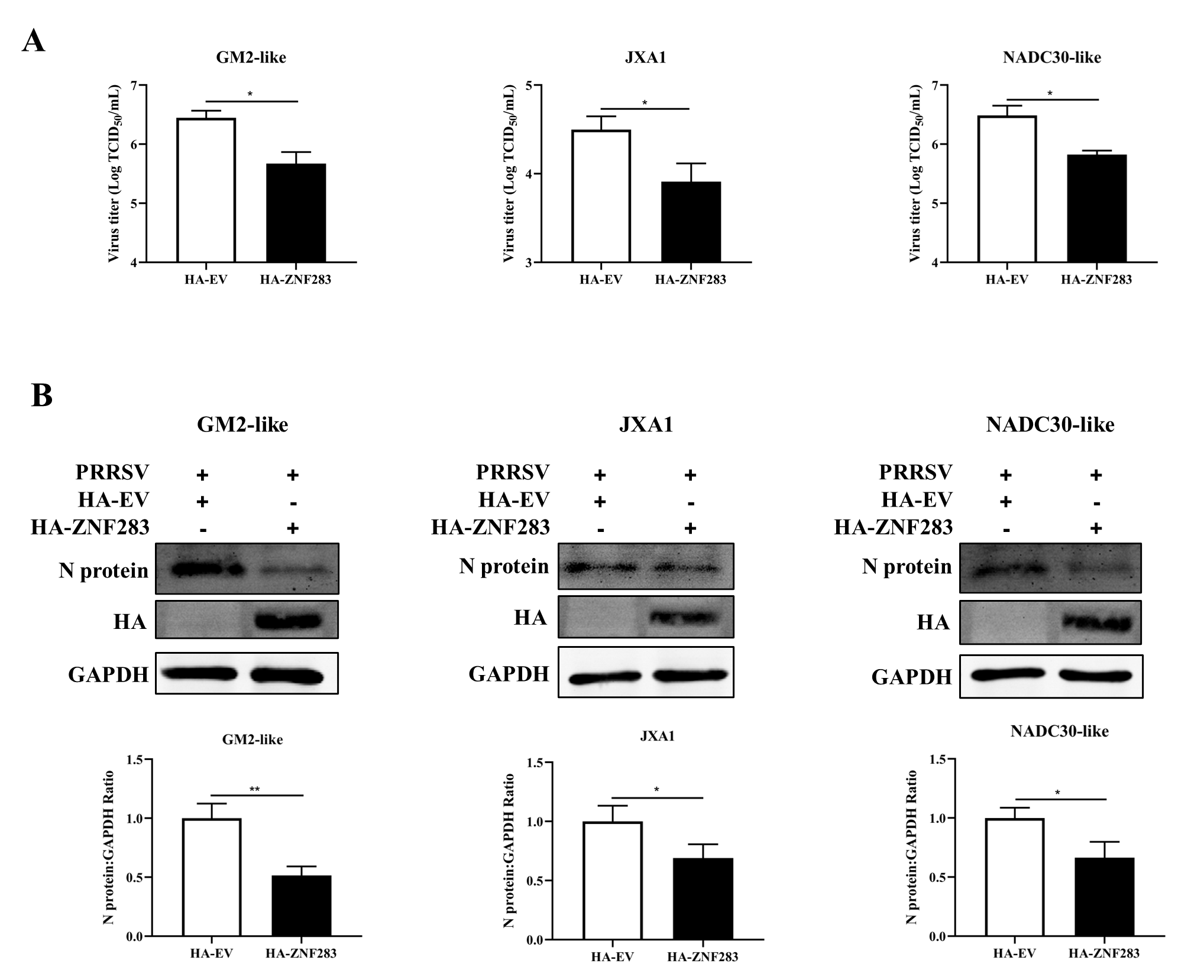

Supplement: Supplementary file 2 — Additional file 2. ZNF283 inhibits the replication of different PRRSV strains. Marc-145 cells were transfected with HA-tagged empty vectors or HA-tagged ZNF283 for 24 h and then infected with different PRRSV strains (GM2-like, JXA1, and NADC30-like) at an MOI of 0.2. At 36 h post-infection, viral titres in the cell supernatant (A) and the expression levels of the N protein (B) were assessed using TCID50 and Western blotting, respectively. [file 13567_2023_1263_MOESM2_ESM.docx]

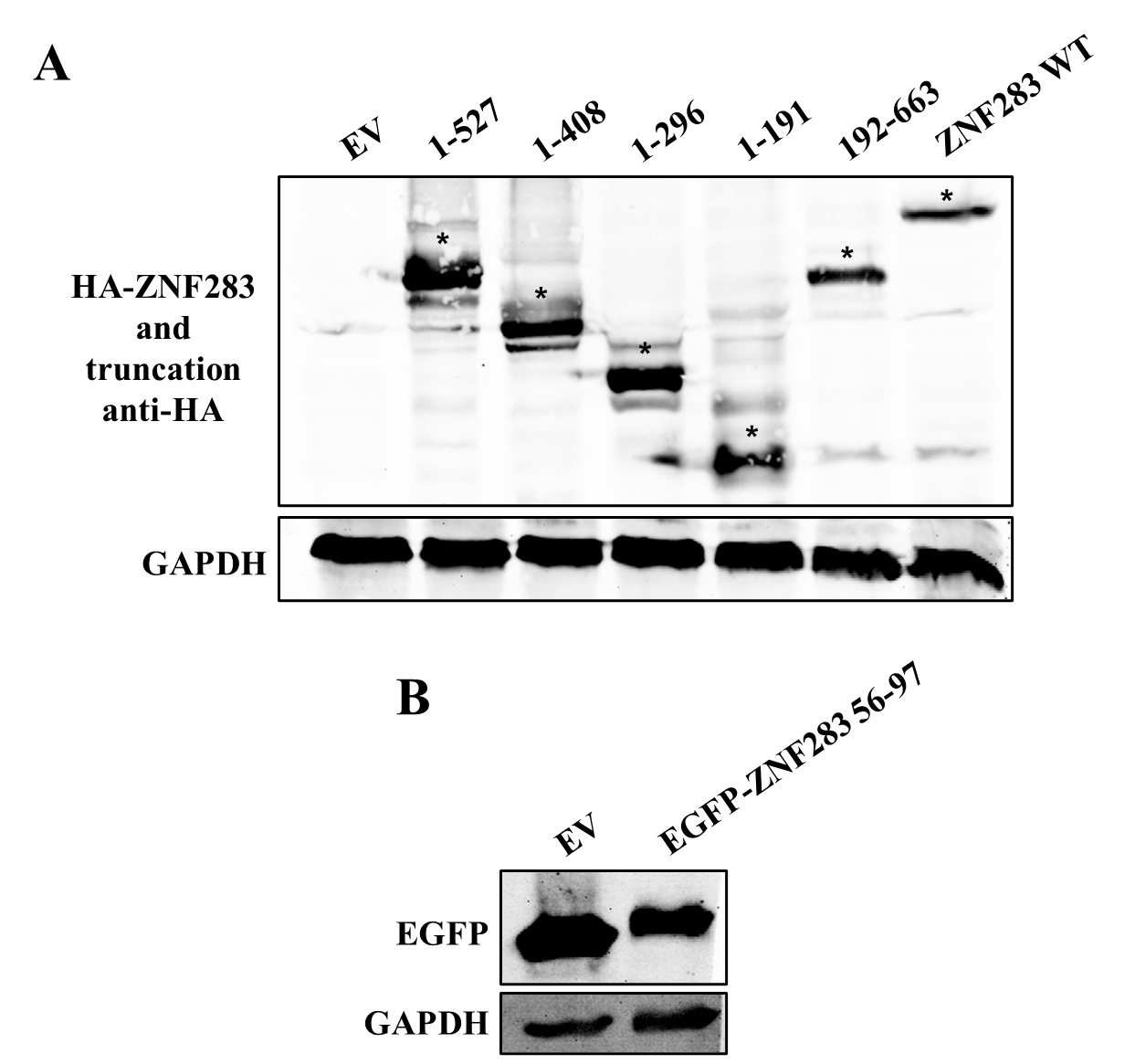

Supplement: Supplementary file 3 — Additional file 3. Verification of the expression of ZNF283 truncation mutants. HEK-293T cells were transfected with plasmid-encoded HA-tagged full-length and truncated ZNF283 (aa 1–191, 1–296, 1–408, 1–520, and 192–663) (A) or EGFP-tagged ZNF283 aa 56–97 (B) for 36 h. Cell lysates were analysed via Western blotting using rabbit anti-HA or rabbit anti-EGFP. [file 13567_2023_1263_MOESM3_ESM.docx]

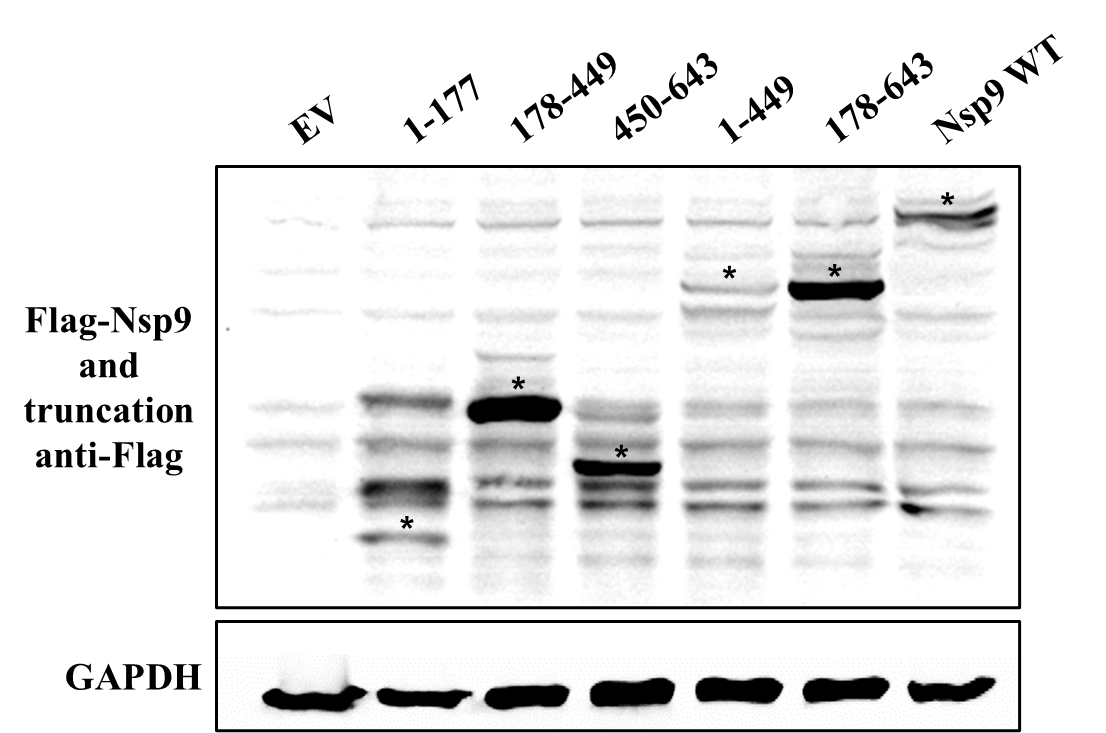

Supplement: Supplementary file 4 — Additional file 4. Verification of the expression of Nsp9 truncation mutants. HEK-293T cells were transfected with empty vector or plasmid-encoded Flag-tagged full-length or truncated Nsp9 (aa 1–177, 178–449, 450–643, 1–499, or 178–643) for 36 h. Cell lysates were analysed via Western blotting using mouse anti-Flag. [file 13567_2023_1263_MOESM4_ESM.docx]
